# Supplementary material for: Splice-Junction-Based Mapping of Alternative Isoforms in the Human Proteome
Source: Cell Rep. Author manuscript; Available in PMC 2020 Jan 15. (PMC6961840; doi:10.1016/j.celrep.2019.11.026)
Supplement: 2 [file NIHMS1546469-supplement-2.zip › DF1/PXD006675/LeftCoronaryArtery/LeftCoronaryArtery_13_MYBPC3_RTDSHEDTGILDFSSLLK.pdf]

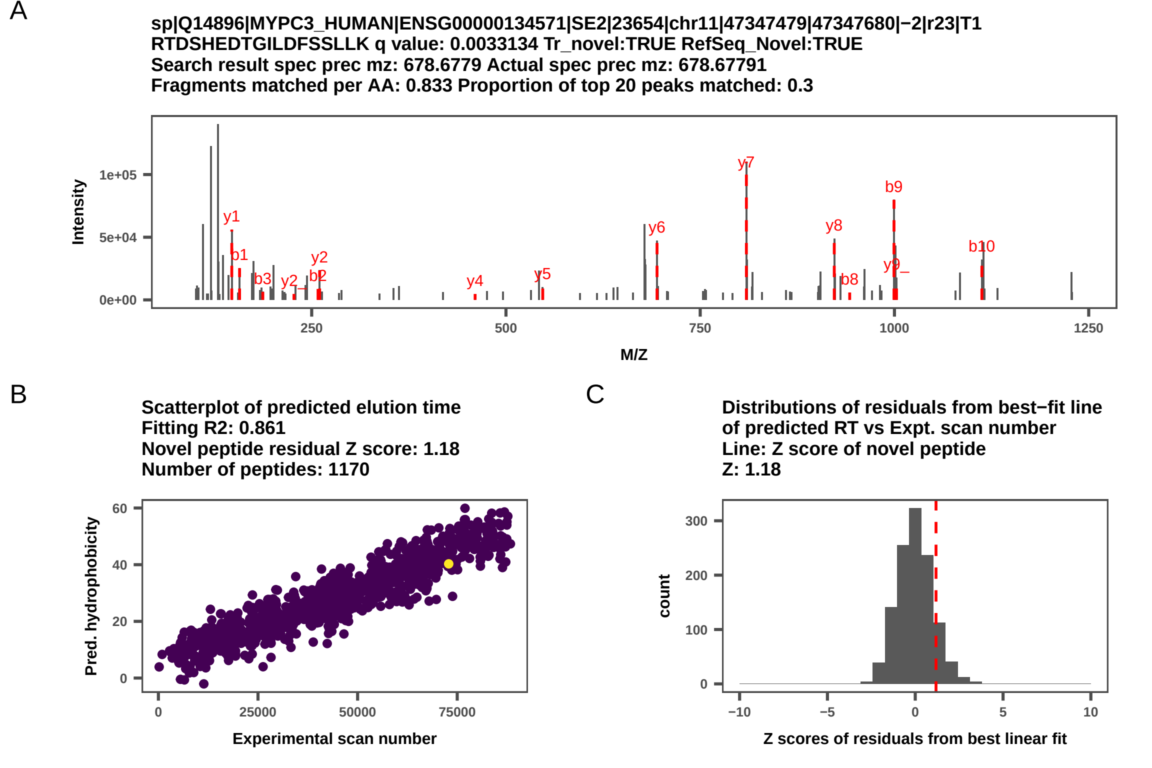
A
sp|Q14896|MYPC3_HUMAN|ENSG00000134571|SE2|23654|chr11|47347479|47347680|−2|r23|T1
RTDSHEDTGILDFSSLLK q value: 0.0033134 Tr_novel:TRUE RefSeq_Novel:TRUE
Search result spec prec mz: 678.6779 Actual spec prec mz: 678.67791
Fragments matched per AA: 0.833 Proportion of top 20 peaks matched: 0.3
Intensity
1e+05
5e+04
0e+00
y1
b1
b3
y2_
y2
b2
y4
y5
y6
y7
y8
b8
b9
y9_
b10
250
500
750
1000
1250
M/Z
B
Scatterplot of predicted elution time
Fitting R2: 0.861
Novel peptide residual Z score: 1.18
Number of peptides: 1170
Pred. hydrophobicity
60
40
20
0
0
25000
50000
75000
Experimental scan number
C
Distributions of residuals from best−fit line
of predicted RT vs Expt. scan number
Line: Z score of novel peptide
Z: 1.18
count
300
200
100
0
−10
−5
0
5
10
Z scores of residuals from best linear fit
